# Pharmaceutical Residues Affecting the UNESCO Biosphere Reserve Kristianstads Vattenrike Wetlands: Sources and Sinks

**DOI:** 10.1007/s00244-016-0303-7

**Published:** 2016-08-01

**Authors:** Erland Björklund, Ola Svahn, Søren Bak, Samuel Oppong Bekoe, Martin Hansen

**Affiliations:** 1 Division of Natural Sciences, School of Education and Environment, Kristianstad University, 291 88 Kristianstad, Sweden; 2 Analytical Biosciences, Department of Pharmacy, Faculty of Health and Medical Sciences, University of Copenhagen, 2100 Copenhagen, Denmark; 3 Department of Pharmaceutical Chemistry, Faculty of Pharmacy and Pharmaceutical Sciences, Kwame Nkrumah University of Science and Technology, Kumasi, Ghana; 4 Department of Environmental and Civil Engineering, University of California, Berkeley, CA 94720 USA; 5 Department of Integrative Biology, University of California, Berkeley, CA 94720 USA

## Abstract

This study is the first to investigate the pharmaceutical burden from point sources affecting the UNESCO Biosphere Reserve Kristianstads Vattenrike, Sweden. The investigated Biosphere Reserve is a >1000 km^2^ wetland system with inflows from lakes, rivers, leachate from landfill, and wastewater-treatment plants (WWTPs). We analysed influent and treated wastewater, leachate water, lake, river, and wetland water alongside sediment for six model pharmaceuticals. The two WWTPs investigated released pharmaceutical residues at levels close to those previously observed in Swedish monitoring exercises. Compound-dependent WWTP removal efficiencies ranging from 12 to 100 % for bendroflumethiazide, oxazepam, atenolol, carbamazepine, and diclofenac were observed. Surface-water concentrations in the most affected lake were ≥100 ng/L for the various pharmaceuticals with atenolol showing the highest levels (>300 ng/L). A small risk assessment showed that adverse single-substance toxicity on aquatic organisms within the UNESCO Biosphere Reserve is unlikely. However, the effects of combinations of a large number of known and unknown pharmaceuticals, metals, and nutrients are still unknown.

Micropollution of aquatic habitats due to anthropogenic activities is considered one of the major future environmental challenges because it may lead to adverse effects on a number of species (Schwarzenbach et al. 2006), and even cause a decrease in species richness and evenness of entire habitats (Johnston and Roberts 2009). Especially the mixture of compounds that work in concert, such as the release of a multitude of pharmaceuticals into the environment, has received increased attention lately (Celander 2011; Galus et al. 2013) due to the still relatively unknown effects on biota and ecosystems. We recently reported on the occurrence and the principal environmental pollution pathways of pharmaceuticals on the well-known but vulnerable Balearic Island Mallorca (Spain), which is visited by 14 million tourists each year (Rodríguez-Navas et al. 2013). Here wastewater-treatment plant (WWTP) effluents containing pharmaceutical residues ended up in marine water bodies, whereas leaching from landfills was a minor source of pharmaceuticals to groundwater aquifers. On Mallorca, measures are necessary to protect groundwater, which is used as drinking water, but it is also important to conserve aquatic environments for coming generations because contamination of such habitats may cause chronic stress, altered behaviour, and, in the worst case, extinction of wild animal populations including fish (Kidd et al. 2007; Brodin et al. 2013; Arnold et al. 2013; Bean et al. 2014). Consequently, the deterioration of such environments is a threat to both the fishing industry and tourism and therefore would have a direct consequence to the income of hundreds of thousands of Mallorquin people if a decrease is seen in the number of visitors.

Now, for the first time, we report on pharmaceutical sources and sinks influencing an ecologically unique area called the Biosphere Reserve Kristianstads Vattenrike (Vattenriket for short). The biosphere is 1040 km^2^ (Fig. 1) and located in the southern most part of Sweden, Region Skåne, and was the first Biosphere Reserve established in Sweden (in 2005). Only five Biosphere Reserves in Sweden are officially recognized by the United Nations agency UNESCO and run under the UNESCO program Man and Biosphere (Kristianstad Vattenrike MAB 2015). This system of open-water bodies covers most of the municipality of Kristianstad including inland forests and water systems all of the way to Hanöbukten bay with rich wetlands lining the largest river in the region, Helge Å, thus creating a large number of habitats. At least 38 fish species have been found in the waters of this truly unique area (Vattenriket Kristianstad Municipality 2015).

**Fig. 1:**
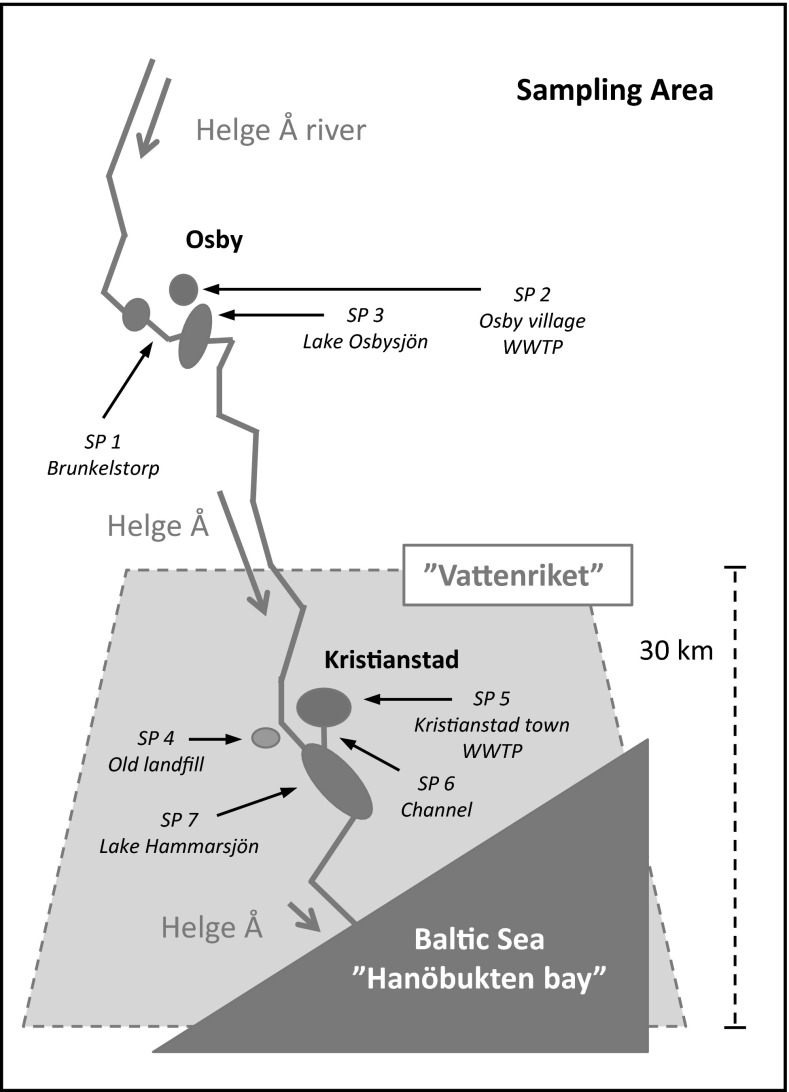
Schematic overview of sampling points (SP1 through SP7) from the water system connected to the UNESCO Biosphere Reserve Kristianstads Vattenrike, Sweden

The sedimentary rocks of the Kristianstad plain hosts Sweden’s (and possibly one of northern Europe’s) largest aquifers, which is also a very valuable natural resource. Large parts of this aquifer lie within the Biosphere Reserve. According to the UNESCO program Man and Biosphere (UNESCO MAB 2015) the aim of establishing a Biosphere Reserve is *“*to set a scientific basis for the improvement of the relationships between people and their environment globally. Launched in the early 1970s, the MAB Programme proposes an interdisciplinary research agenda and capacity building that target the ecological, social and economic dimensions of biodiversity loss and the decrease of this loss.” As a Biosphere Reserve, Kristianstad municipality should be at the forefront of handling the negative impact of anthropogenic effects on the environment.

The Hanöbukten bay is a major touristic area during summer with 40 km of mainly sand beaches. Therefore, ecosystem services—such as opportunities for recreation in nature, swimming, sport fishing, and local food production—play a key factor in keeping this eastern part of the region economically sustainable. However, a recent report by The Swedish Agency for Marine and Water Management (Hanöbukten 2013) concluded that observations made by local fishermen and the public reveals that parts of Hanöbukten intermittently experience problems with decreased incidence of fish and wounded fish. Unfortunately, an in-depth analysis of the area has not been able to identify a single factor or source as the underlying cause. However, a number of knowledge gaps have been identified including a lack of data on the water chemical burden including that of pharmaceutical residues. The report speaks of the so-called “cocktail effect”, i.e., a cocktail partly consisting of pharmaceuticals, which must be better understood to decrease the risk of additive effects on biota.

Pharmaceutical compounds are small- to medium-sized organic molecules with differing hydrophobicity and diverse functional groups attached to the main hydrocarbon skeleton (Barron et al. 2008). This leads to pharmaceuticals covering the full spectra from acidic through neutral to basic compounds as well as varying degrees of hydrophobicity. In this study, six compounds were analysed including atenolol, bendroflumethiazide, carbamazepine, diclofenac, furosemide, and oxazepam, which cover much of the variation seen among various pharmaceuticals (Fig. 2). We recently published an in-depth sorption study of the six pharmaceuticals to the very same four natural sediment samples in this study (Svahn and Björklund 2015). It was shown that sorption effects, measured as asymmetry factors and recoveries, differed pronouncedly among the pharmaceuticals and between the matrices, which could be explained by the basic physicochemical properties of the investigated compounds in relation to matrix characteristics. Protonated and deprotonated molecular properties had the greatest importance for sorbate–sorbent interactions. Such knowledge may aid in understanding how the six selected pharmaceuticals distribute within *Vattenriket*.

**Fig. 2:**
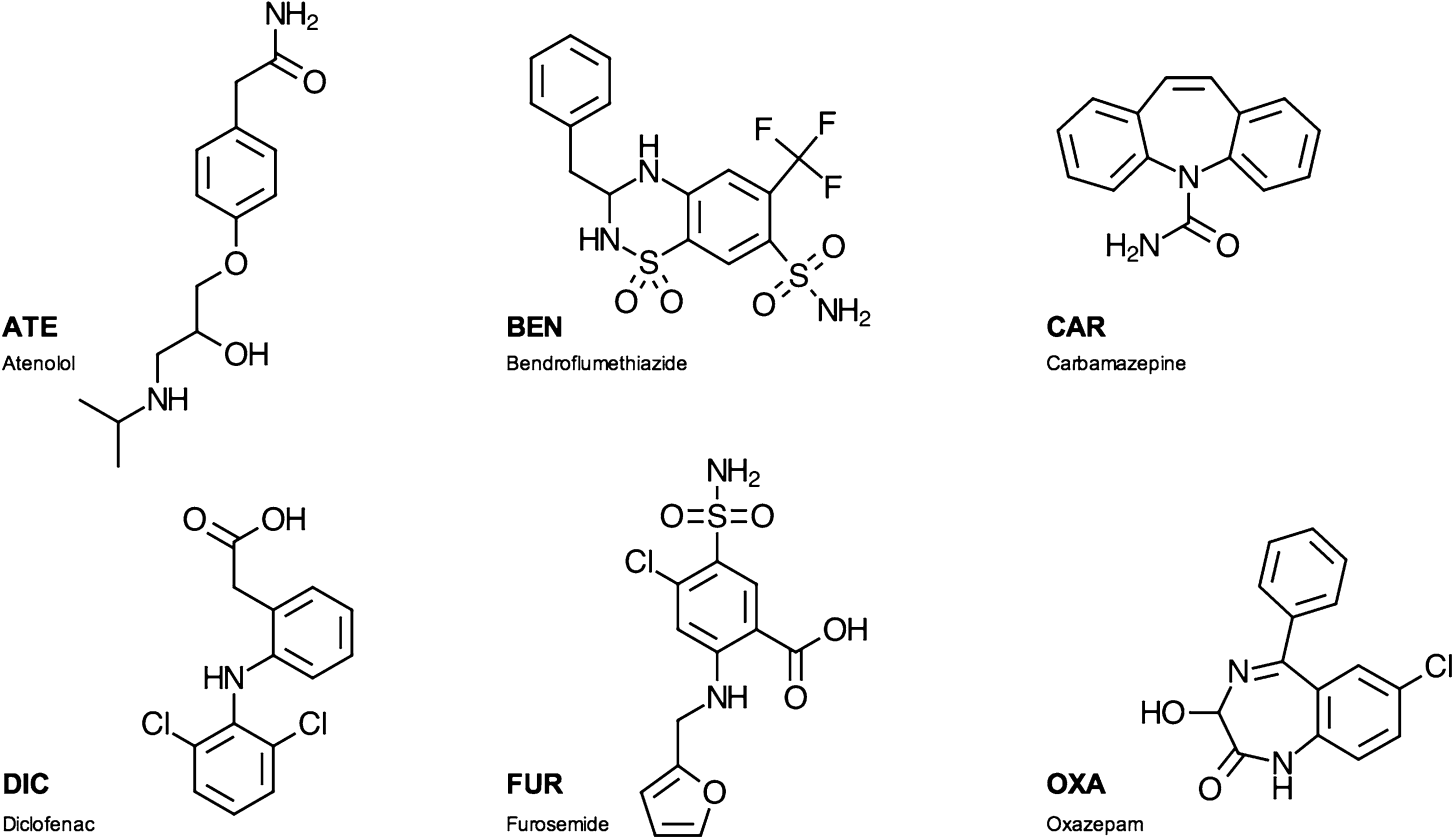
Molecular structures of the six investigated pharmaceutical compounds: *ATE* atenolol, *BEN* bendroflumethiazide, *CAR* carbamazepine, *DIC* diclofenac, *FUR* furosemide, and *OXA* oxazepam

Apart from chemical characteristics, the choices of molecules were also based on pharmaceutical consumption in Region Skåne in the south of Sweden, thus representing the smallest geographical unit of available statistics from where the water and sediment samples were collected. Statistics are available from the Swedish National Board of Health and Welfare (Socialstyrelsen 2015) describing drug consumption on an annual basis. By using the measure defined daily dose (DDD), it is possible to derive a measure that indicates the amount of consumed substance in an area where DDD for a drug is the assumed average maintenance dose per day for a drug used for its main indication in adults (WHOCC 2015).

Thus far no complete evaluation of the economical losses caused by a decrease in fish populations in *Vattenriket* and the Hanöbukten bay has been performed. However, a number of estimations of the monetary values of tourism and fishing industry have been performed (Hanöbukten 2013) showing that the landed value of fish exceeds 20 million SEK, whereas tourism as a whole is worth hundreds of million SEK.

The objective of this study was to gain knowledge on the chemical burden of pharmaceutical micropollutants upstream and inside the *Vattenriket* water system. We aim to identify (1) the pharmaceutical sources and sinks; (2) the possible relationships between the local drug-consumption pattern and the measured concentrations; and finally (3) show if any of the surface-water concentrations exceed adverse effect levels on aquatic organism. Such information is crucial for local authorities to take action to improve and conserve the great fisheries and tourism values in the area.

## Experimental

### Study Sites and Sampling

As Helge Å flows toward *Vattenriket*, the chemical nature of the river shifts from a humus-rich soft water (pH 6.3 at Brunkelstorp and Lake Osbysjön) to slightly alkaline water (pH 7.8 at Lake Hammarsjön) as described later in the text (Fig. 1). Our first sampling point (SP1) is located at Brunkelstorp, which is a section of the River Helge Å upstream of Lake Osbysjön and the Osby village. At SP1 surface water and sediment was sampled. The WWTP of Osby village (SP2), serving 12,000 people and including biological and chemical treatment steps followed by sedimentation, releases its treated water into lake Osbysjön (SP3). Inlet and effluent water was sampled at SP2 WWTP, whereas lake water and sediment was collected at SP3. Helge Å River then flows toward Kristianstad town. At the heart of *Vattenriket*, in close proximity to the Helge Å and Kristianstad city centre, lays the old landfill of Kristianstad (Härlövsdeponin). The landfill was closed in 2002 and was covered with a 1.5 m protective layer during the years 2011 to 2014. Old landfill leachate samples were collected at SP4. This leachate is presently pumped to the WWTP of Kristianstad town treating wastewater corresponding to 150,000 personal equivalents with a majority coming from industries and the central hospital where 55,000 individuals are connected. The WWTP is one of the largest in Sweden and uses biological and chemical treatment steps as well as sedimentation and final polishing through sand. The treated wastewater of Kristianstad town (SP5) flows into a 1500 m channel (SP6), which is characterized by slowly flowing water. WWTP inlet and effluent water was sampled at SP5, whereas surface water and sediment were collected at SP6. The channel water is then pumped into Lake Hammarsjön (SP7). Lake water and sediment was collected at SP7. Noteworthy is that the WWTP of Kristianstad town is located close to the lowest point in Sweden (2.5 m below sea level) which is the reason for the channel water being pumped 2 m upward into lake Hammarsjön. At each sampling point, both surface water and sediment samples were collected in glass bottles and chemically characterized. All samples were transported to the laboratory in cooled boxes within 3–4 h after collection and further stored in a freezer at −22 °C and processed as described in later sections of the text. Technical personnel at the two WWTPs collected inlet- and outlet-water samples. At both Osby and Kristianstad, WWTP grab samples were taken, whereas at the Kristianstad site a complementary 24 h sample was made available. All samples were collected as single-grab samples at each sampling point. All water samples were collected in glass bottles thoroughly cleaned with soap, further flushed three times with Milli-Q quality water (Millipore) and 96 % ethanol (Kemethyl, Denmark), and dried at 150 °C before sampling. Using a time-integrated sampler, 24 h samples were taken by the personnel at the WWTPs. Sediment samples were taken by a special bottom-sampler, Ekmanhuggare, taking out a portion of the top few centimetres of the sediment.

### Chemicals and Standards

Atenolol, bendroflumethiazide, carbamazepine, diclofenac, and furosemide were obtained from Sigma-Aldrich (Steinheim, Germany). Oxazepam was obtained from Apoteket (Kristianstad, Sverige). Stock solutions of the individual pharmaceuticals in methanol were prepared at a concentration of 10 mg L^−1^ and stored at −18 °C. Deuterated internal standards (ISs), d_10_-carbamazepine and d_7_-atenolol, were obtained from CDN Isotopes (Pointe-Claire, Quebec, Canada). The purity of all analytical standards was >95 % and deuterated purity was >98 %. Ammonium acetate (98 %) was obtained from KMF (St. Augustin, Germany) and acetic acid (99–100 %), aqueous ammonia solution, heptane, ethyl acetate, methanol, and acetone from Merck (Darmstadt, Germany). Ethylenediaminetriacetic acid (EDTA) was obtained from Sigma-Aldrich (>98 %). Ammonium formate, formic acid, acetonitrile, and water were purchased from Merck.

### Chemical Analysis

Chemical analysis followed our previously validated solid-phase extraction (SPE)—liquid chromatography (LC)—electrospray ionization (ESI)—tandem mass spectrometry (MS/MS) protocol for the determination of pharmaceutical residues (Pérez-Carrera et al. 2010).

#### Water Samples—SPE

Before SPE, samples were filtered using Whatman 0.45-m porous GF/C fibre disks conditioned with 500 mg EDTA/L of sample and pH adjusted to 7.0 with acetic acid (>99 %; J. T. Baker, Holland). Only the water phase was further analyzed. Hereafter, the water samples were fortified with 500 ng of each of the ISs. SPE cartridges were kept in darkness at −22 °C. Clean-up and preconcentration of both water samples and sediment extracts was performed using SPE using a combination of MAX-cartridges (anion exchanger 150 mg, 6 mL) and HLB-cartridges (hydrophilic–lipophilic balance 200 mg sorbent, 6 mL cartridge) purchased from Waters Oasis (Milford, Massachusetts, USA). The Vacumaster-manifold for the SPE cartridges was obtained from IST (Glamorgan, UK). Briefly, both cartridges were preconditioned with 5 mL of heptane, 5 mL of ethyl acetate, 5 mL of methanol, and 2 × 5 mL of tap water (pH 7.0 adjusted). A MAX cartridge was then placed on top of the HLB cartridge with a polytetrafluoroethylene adaptor and placed in the SPE manifold vacuum system. The samples were passed through both cartridges at an approximate flow rate of 5 mL min^−1^. Subsequently the HLB and MAX cartridges were disassembled and air-dried for 30 min. Both SPE cartridges were washed with 5 mL of heptane. Finally, the MAX cartridge was eluted with 1 mL of ethyl acetate, 2 mL of methanol, 2 mL of methanol containing 2 % acetic acid, and 2 mL of methanol; the HLB cartridge was eluted with 3 mL of ethyl acetate and 4 mL of methanol. These two aliquots from MAX and HLB were combined, divided in two, and evaporated to dryness at 60 °C under a gentle stream of nitrogen. Each of the combined eluates was reconstituted in 200 µL of a mobile phase mix A and B (1:1, v/v) from high-performance liquid chromatography (HPLC) and transferred into two HPLC vials with 300 µL glass inserts.

#### Sediment Samples—Pressurized Hot-Water Extraction

Pharmaceuticals were extracted from freeze-dried sediment samples using a homemade apparatus for subcritical and supercritical water extraction similar to the one described by Hawthorne et al. (1994). The outlet water flow pressure was controlled by an SSI high-pressure needle two-way valve (Grace Discovery Sciences) with pressure held at 20 bars during experimental runs. Before the needle valve, a 1 m coil [1/16″ outer diameter, 0.020″ inner diameter (ID)] was mounted and air-cooled using a common electrical fan (12 V), thus assuring that the temperature of the water was <25 °C. Temperature was monitored with thermocouples at the outlet from the gas chromatography oven and after the needle valve. The extraction cell consisted of a stainless steel column with a volume of 11.8 mL (150 × 10.0 mm ID). The cell was carefully packed with a mixture of 0.5 g sample and glass beads measuring 1 mm in diameter. A punched glass-filter (GF/C) was added to the column outlet. The column was connected and put into the oven, the pump turned on, and the temperature set to 150 °C. Approximately 200 mL of water at a flow rate of 2 mL/min was used to extract the analytes. Thereafter, the sediment extracts were purified using the SPE protocol described previously.

#### LC-MS/MS

Analysis of the resulting extracts was performed according to LC-ESI-MS/MS method I (Pérez-Carrera et al. 2010). Using 5-µL injections, HPLC separation was achieved by a reversed-phase column (XTerra MS-C18, 2.1 × 100 mm, 3.5 µm; Waters Oasis). The analytical system consisted of an Agilent 1100 series HPLC system (Agilent, Palo Alto, California, USA) equipped with a degasser, a cooled autosampler (4 °C), and a column oven (30 °C). Detection was performed using a Sciex API 3000 triple-quadrupole mass spectrometer (Applied Biosystems, Foster City, California, USA) equipped with an ESI source (Turbo Ion spray). For MS/MS detection, the instrument was operated in SRM mode in combination with multiple polarity (switching) mode. Comparing retention times and substance-specific mass spectra, positive identifications were achieved. Precursor ions and product ions and other LC-MS/MS parameters are listed in the work by Pérez-Carrera et al. (2010). Collection and treatment of data were performed using Analyst 1.4 software (Applied Biosystems) in a Windows XP platform-based data-processing system.

## Results and Discussion

### Region Skåne Consumption Pattern and WWTP Inlet-Water Concentrations

**Fig. 3:**
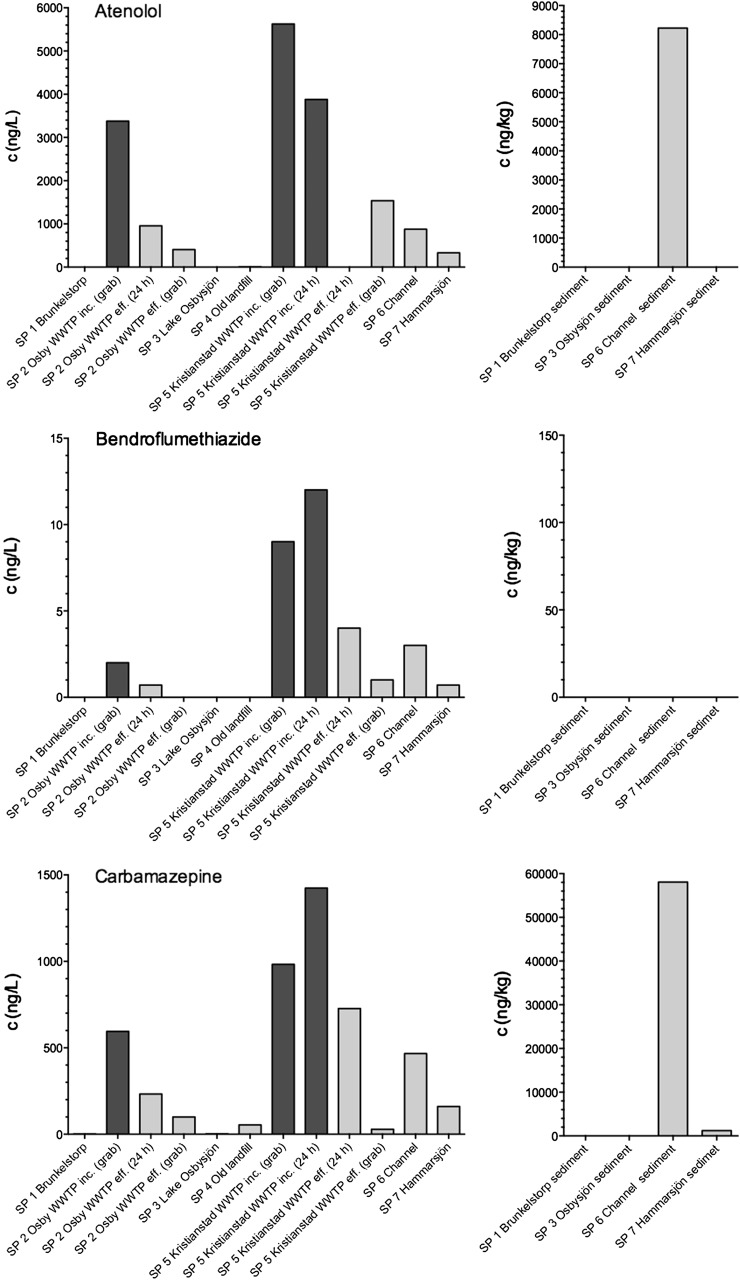
Measured concentrations of atenolol, bendroflumethiazide, and carbamazepine in aqueous samples and sediment samples (dry weight) at the various sampling sites. The results are displayed in the order SP1 to SP7 representing the flow direction of the water through the various sampling sites. Sampling sites are shown in Fig. 1. *Left panel dark grey bars* represent incoming water, and *light grey bars* represent effluent or surface waters

**Fig. 4:**
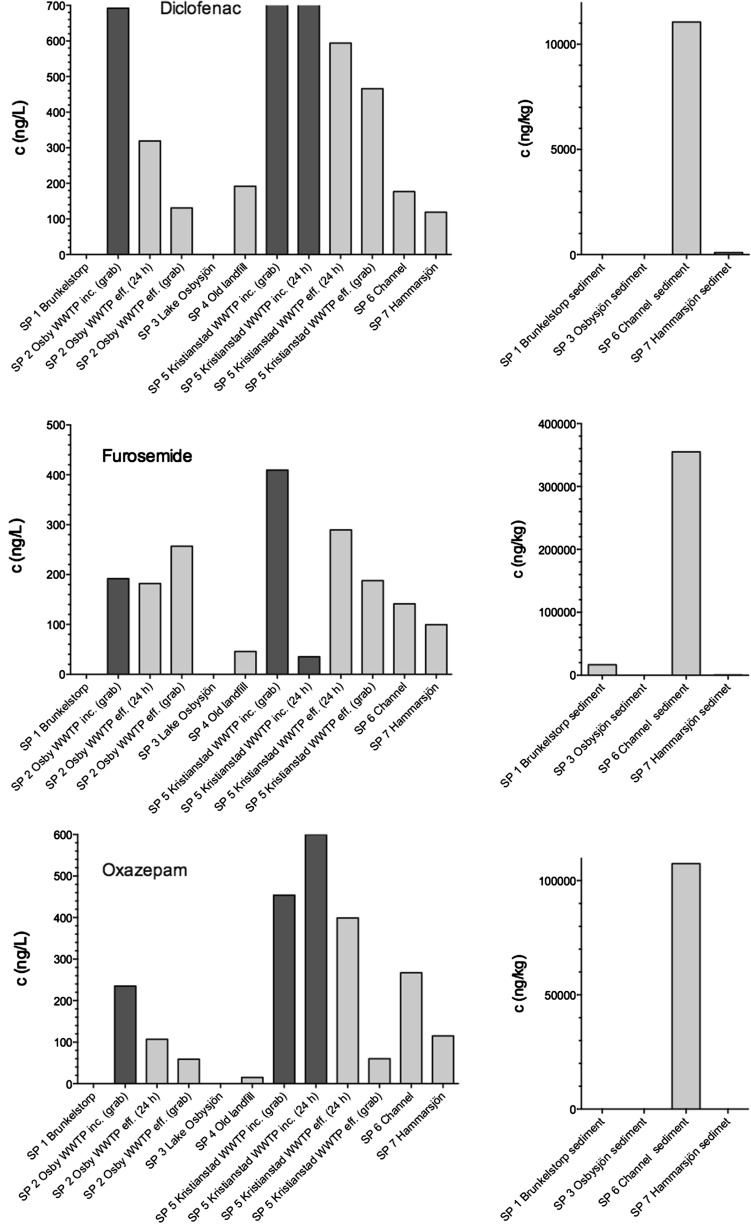
Measured concentrations of diclofenac, furosemide, and oxazepam in aqueous samples and sediment samples (dry weight) at the various sampling sites. The results are displayed in the order SP1 to SP7 representing the flow direction of the water through the various sampling sites. Sampling sites are shown in Fig. 1. *Left panel dark grey bars* represent incoming water, and *light grey bars* represent effluent or surface waters

The calculated total amount of drug administered for the six compounds is listed in Table 2. The least consumed compounds (kg/y basis) were the antihypertensive agent bendroflumethiazide (27 kg) and the anxiolytic compound oxazepam (87 kg). This was followed by the β-blocker atenolol (450 kg), the antihypertensive agent furosemide (720 kg), and the antiepileptic drug carbamazepine (721 kg). Finally, the most used pharmaceutical was the nonsteroidal anti-inflammatory compound diclofenac (897 kg). Based on the information in Table 2, the consumption pattern of pharmaceuticals in Region Skåne could now be related to the concentrations at the various sampling points. The results from the chemical analysis of samples at the various sites are shown in Fig. 3 (atenolol, bendroflumethiazide, and carbamazepine) and Fig. 4 (diclofenac, furosemide, and oxazepam). The results are displayed in the order SP1 to SP7 representing the flow direction of the water through the various sampling sites. The incoming concentrations of the six selected pharmaceuticals are shown in dark grey bars. At both Osby and Kristianstad, WWTP grab samples were taken, whereas at the Kristianstad site a complementary 24 h sample was made available. All investigated compounds were detected in the grab samples at both Osby and Kristianstad WWTPs (Figs. 3, 4) ranging from a few ng/L of bendroflumethiazide to several thousand ng/L of atenolol.

According to the low value of consumption data for bendroflumethiazide (27 kg), it was reasonable to expect that this compound would be found at the lowest levels in the incoming water, and the assumption proved accurate (Fig. 3) showing concentrations of ≤12 ng/L. In a study performed at two Swedish WWTPs in the area of Stockholm during the years 2005–2009, the average incoming concentrations were 20 ng/L (Henriksdal, population 720,000) and 10 ng/L (Bromma, population 300,000), respectively, which was very close to our data (Stockholms Vatten Report). In the human body, bendroflumethiazide is readily metabolised, and only 30 % is excreted unchanged in the urine whereas the remainder is excreted as uncharacterized metabolites, which might further support the low incoming concentrations in relation to, e.g., oxazepam as discussed later in the text (Electronic Medicines Compendium 2015).

Oxazepam was the second lowest consumed drug, three times greater than bendroflumethiazide. Yet, the incoming concentrations were 50–100 times greater than for bendroflumethiazide, *i.e.*, in the range 200 and 600 ng/L (Fig. 4). The influent concentrations were very close to those reported from two Swedish sites in Stockholm with values of 338 and 223 ng/L for Henriksdal and Bromma, respectively (Stockholm Vatten 2010). Similar concentrations were reported in another Swedish survey of WWTPs (IVL 2011). The survey covered 4 sites with 57,000 (Skövde), 100,000 (Umeå), 160,000 (Uppsala), and 835,000 (Stockholm) people connected to them. Inlet waters contained 220–310, 280–390, 470–1800, and 360–460 ng/L, respectively. In two recent studies from Cĕské Budĕjovice (Czech Republic, 112,000 inhabitants [Golovko et al. 2014]) and Rouen (France, 400,000 inhabitants [Saussereau et al. 2013]), median measured concentrations were 56 and 1160 ng/L, respectively. This shows the widespread occurrence of this compound in European WWTPs as well as the relatively large differences in incoming water concentrations determined in various WWTPs. There are reports of oxazepam being excreted unmetabolised (Stockholm Vatten 2010), thus supporting the high incoming concentrations. In addition, in a recent study oxazepam was shown to be persistent over long periods of time in the environment (Klaminder et al. 2015).

Almost half a ton of atenolol was consumed in Region Skåne, five times more than oxazepam. Correspondingly, the measured concentrations were greater for this compound by a factor of approximately 10 compared with oxazepam, reaching almost 6,000 ng/L at Kristianstad WWTP (Fig. 3). Atenolol is well known to occur at high levels in Sweden (IVL 2011; Stockholm Vatten 2010). The inlet waters in the four previous WWTPs contained 540–780 ng/L (Skövde), 1100 ng/L (Umeå), 1200–4900 ng/L (Uppsala), and 330–590 ng/L (Stockholm), whereas Henriksdal and Bromma had concentrations of 1470 and 1390 ng/L, respectively. A finish study by Vieno et al. (2006) at three WWTPs serving populations of 12,400, 27,000, and 40,000 people had inlet concentrations of 510, 750, and 800 ng/L, respectively. In a Spanish study by Rosal et al. (2010) covering the area of Alcalá de Henares, Madrid (population 374,000), samples were taken once a month for a period of 1 year. Here the average atenolol influent concentration was 1197 ng/L with minimum and maximum concentrations of 660 and 2432 ng/L, respectively. Finally, in a study from Betzdorf (10,000 PE, Luxembourg), the median concentration only reached 186 ng/L during a 9-day period (Majewsky et al. 2013). Overall it is not uncommon that atenolol concentrations exceed 1 μg/L in Sweden and throughout Europe, although there is substantial variation in inlet concentrations. These high inlet concentrations are likely caused by atenolol passing through the human body without being completely metabolized (Wadworth et al. 1991; Vieno et al. 2006). Based on a literature survey, Kasprzyk-Hordern et al. (2007, 2008) concluded that atenolol was excreted up to approximately 50 % in unchanged form, whereas others report values as high as 90 % (Stockholm Vatten 2010).

However, high consumption did not necessarily mean that levels in the incoming water were high. Furosemide, despite being consumed 8 times more than oxazepam, still showed levels very close to those of oxazepam with concentrations of a few hundred ng/L (Fig. 4). This was true at both Osby and Kristianstad WWTP. The incoming grab sample of Kristianstad exceeded 400 ng/L, which is almost identical to the Spanish 1-year study mentioned previously by Rosal et al. (2010) that had an average value of 413 ng/L. Somewhat greater inlet concentrations were found in Sweden 1550 ng/L (Henriksdal) and 1200 ng/L (Bromma) (Stockholm Vatten 2010). One possible explanation for the relatively lower concentration levels might be that only minute amounts of ingested furosemide are excreted in an unchanged form as proposed by Kasprzyk-Hordern et al. (2008). However, in sharp contradictions stands a Swedish report (Stockholm Vatten 2010) stating 90 % unaltered excretion, thus making it difficult to draw any certain conclusions concerning furosemide.

Carbamazepine showed equal consumption compared with furosemide, whereas the incoming water concentrations were 2 to 3 times greater, i.e., on the order of 500–1500 ng/L (Fig. 3). The compound has been observed at similar levels in several Swedish WWTPs (IVL 2011). The inlet waters had concentration of 380–560 ng/L (Skövde), 1100–1600 ng/L (Umeå), 580–2600 ng/L (Uppsala), and 370–560 ng/L (Stockholm). Likewise, Henriksdal and Bromma had concentration levels on the same order of magnitude, i.e., 300 and 275 ng/L, respectively (Stockholm Vatten 2010). In the Spanish study mentioned previously by Rosal et al. (2010) the average inlet water concentration was 129 ng/L, which is lower than observed in our study. Somewhat greater concentrations were seen in the French study by Saussereau (2013) showing median daily measured concentrations of 510 ng/L, which is close the concentrations found at Osby WWTP. Likewise, in the study from Luxembourg the median concentration was 429 ng/L (Majewsky et al. 2013). Overall the consumption and inlet water concentrations show a fairly high degree of similarity between furosemide and carbamazepine. This also fits with the reported unchanged excretion percentages, which have been declared to be 2–3 % for both compounds (Kasprzyk-Hordern et al. 2008; Sui et al. 2010). It should be noted, however, that there are reports on as much as 28 % of the compound being excreted in unchanged form by way of faeces (Zhang et al. 2008).

Finally, the consumption of diclofenac reached almost 900 kg, which is approximately 25 % greater than carbamazepine and furosemide (Fig. 4). The influent concentrations of this compound were very stable, approximately 700 ng/L at both Osby and Kristianstad WWTP, and somewhere in between those concentrations observed for carbamazepine and furosemide. The Swedish WWTPs (IVL 2011) showed concentrations of 120–500 ng/L (Skövde), 970–2800 ng/L (Umeå), 1200–7000 ng/L (Uppsala), and 900–1800 ng/L (Stockholm), whereas our results are at the lower end of these levels. However, even lower values were observed in Sweden where Henriksdal had inlet concentrations of 384 ng/L and Bromma 285 ng/L (Stockholm Vatten 2010). Relatively low concentrations were also reported in the Spanish study by Rosal et al. (2010) with average values of 232 ng/L, whereas the Luxembourg study reported a median concentration of 691 ng/L (Majewsky et al. 2013), which is very close to our data. Diclofenac is highly consumed and also present at measureable levels in various European WWTP studies but with great variation in inlet water concentrations. Sui et al. (2010) reported excretion rates of 15 % as unaltered compound, whereas Zhang et al. (2008) reported that 6 % of the compound was present in urine accompanied by an unknown fraction in faeces (Zhang et al. 2008). Likewise, 1 % excretion was reported in a recent study (Stockholm Vatten 2010). Together these excretion percentages are close to those reported for carbamazepine presented previously as were the inlet concentrations.

### WWTP Pharmaceutical-Removal Efficiency

The removal efficiency of pharmaceuticals is highly variable both between compounds as well as for a single compound at different sites, which is well illustrated in the review by Zhang et al. (2008) covering almost 20 scientific publications spanning a decade. Removal efficiency is often defined as the concentration differences between influent and effluent water divided by the influent concentration. Here we calculated the percent removal efficiency based on the data presented in Figs. 3 and 4. The removal efficiency for atenolol, bendroflumethiazide, carbamazepine, diclofenac, furosemide, and oxazepam were calculated individually for Osby and Kristianstad using the grab samples. Data are presented in Table 3 and compared with removal efficiencies of those reported in the Swedish survey of WWTPs in 2010 (IVL 2011) covering Skövde, Umeå, Uppsala, and Stockholm. Data from the Swedish study at Henriksdal and Bromma during 2005–2009 (Stockholm Vatten 2010) are also presented at the end of Table 3.

The removal of atenolol at both Osby and Kristianstad exceeded 70 % and was similar to the removal efficiencies in Uppsala and Stockholm (Table 3). Yet there seems to be some differences among sites because Skövde and Umeå did not exceed 30 %. Likewise, somewhat lower efficiencies were observed at Bromma with 34 %, whereas Henriksdal had an efficiency value of 55 %. Examples in the literature also show poor removal, *e.g.*, the Spanish study by Rosal et al. (2010) with 15 % removal, whereas in a major survey covering six Italian WWTPs from north to south, the average removal for atenolol was 10 % (range 0–21 %) during winter and 55 % (range 36–76 %) during summer (Castiglioni et al. 2006). A Spanish study including seven WWTPs reported atenolol removal in the range 20 to 97 % with an average of 59 % (±50) (Gros et al. 2010). Although there are large variations in the removal efficiency of atenolol, it can still be concluded that in many cases pharmaceuticals undergo some kind of removal processes. Atenolol carries a positive charge at neutral pH (pKa of 9.6), and in a recent work we showed that atenolol recovery was very low on sludge (Svahn and Björklund 2015). Atenolol has basic properties with a positive charge at pH 7, and a cation exchange probably caused atenolol to bind to the negatively charged organic material of sludge, thereby improving its removal efficiencies.

Bendroflumethiazide was also removed relatively efficiently to between 67 and 100 % (Table 3), but removal efficiency was not determined in the Swedish surveys (IVL 2011; Stockholm Vatten 2010). No other WWTP data were identified with which to compare our results. However, based on our sorption study to sludge, bendroflumethiazide showed a poor recovery and pronounced tailing, thus supporting our observed removal (Svahn and Björklund 2015). In addition, bendroflumethiazide has a special chemical feature in its three fluorine atoms that might increase binding to specific sites of organic materials.

Carbamazepine saw a removal in the range of 48–97 %, whereas the Swedish survey from 2010 showed removal efficiencies of 25 and 33 % in Uppsala and Umeå, respectively (Table 3). Interestingly, at the WWTPs in Skövde and Stockholm, a negative behaviour occurred, although it was not explicitly explained why in the report (IVL 2011). The survey from 2005 to 2009 also showed poor removal <10 % (Stockholm Vatten 2010). Correspondingly, Rosal et al. (2010) reported a 10 % removal, whereas Saussereau et al. (2013) found no removal at all. Comparably, in Italy, Castiglioni et al. (2006) reported no removal of the compound neither in winter nor in summer samples. Remarkably, in a study by Martín et al. (2012) including four WWTPs in Spain, the average removal was close to 50 %, although this had large variations depending on site (−7, 28, 85, and 88 %), thus supporting our findings. Martín et al. (2012) concluded that carbamazepine is a compound with a high chemical stability and that part of its removal from wastewater could be explained by sorption onto sludge due to its semihydrophilic nature. Log P is reported to be 2.45 (Table 1) for this uncharged pharmaceutical. We agree with this explanation based on our sorption study data to sludge with recoveries close to 60 % and with extensive peak tailing (Svahn and Björklund 2015). Despite the large variations observed in the literature, it should be made clear that carbamazepine can most often pass through WWTPs at significant levels and be removed mainly in the range 0–30 % (Zhang et al. 2008), although exemptions to this exist in the literature.

**Table 1:** Name, molecular weight, and physicochemical properties of the six investigated pharmaceutical compounds

Compound	MW (g mol^−1^)	p*K*a	*S*_w_ (mg L^−1^)	log *P*
Atenolol	266.3	9.6	13300	0.16
Bendroflumethiazide	421.4	8.5	108	1.89
Carbamazepine	236.3	–	17.7	2.45
Diclofenac	296.1	4.15	2.37	4.51
Furosemide	330.7	4.25	73.1	2.03
Oxazepam	286.7	–	179	2.24

All values are from DrugBank (2015)

*MW* molecular weight, *pKa* acidity constant, *S*_*w*_ water solubility, *log P* octanol-water partitioning coefficient

**Table 2:** Consumption of the six studied drugs in Region Skåne in 2011

Substance	Number of DDD	1 DDD (mg)	Consumption (kg/years)
Atenolol	6,003,685	75	450
Bendroflumethiazide	10,858,173	2.5	27
Carbamazepine	720,960	1000	721
Diclofenac	8,967,462	100	897
Furosemide	17,994,005	40	720
Oxazepam	1,730,691	50	87

Based on statistics from the National Board of Health (Socialstyrelsen 2015)

*DDD* defined daily dose

Diclofenac also showed varying removal in the range 12–81 % (Table 3). Likewise the Swedish survey showed large variation with removal efficiencies of −27, 23, 50, and 64 % for Skövde, Uppsala, Umeå, and Stockholm, respectively (IVL 2011). In Henriksdal and Bromma, removal efficiencies were at the lower end with values of 20 and 10 %, respectively (Stockholm Vatten 2010). Rosal et al. (2010) observed a 5 % removal, whereas Martín et al. (2012) reported an average removal of 14 % for diclofenac. Martín et al. (2012) suggested that the poor removal of diclofenac (which in many cases even was negative) might be explained by the liberation of additional diclofenac molecules by deconjugation of glucoronidated or sulfated diclofenac and/or its desorption from particles. Gros et al. (2010) reported diclofenac removal in the range 30–100 % with an average of 58 % (±53), which basically covers our reported removal efficiencies. Based on the thorough literature study by Zhang (2008), the removal efficiencies of diclofenac by WWTPs varied but were mainly in the scope of 21–40 %. Zhang et al. (2008) further concluded that sorption behaviour of diclofenac onto sludge is similar to that of carbamazepine although with a greater tendency for diclofenac to bind. Our sorption study points at diclofenac not always being sorbed very firmly by the sludge matrix much due to the negative charge of both sludge organic material and of diclofenac [pKa 4.15 (Table 1)] at neutral pH, thus causing possible repulsion (Svahn and Björklund 2015). In contrast, log P for diclofenac is 4.51 (Table 1), which is substantially greater than that for carbamazepine. Our new data (Svahn and Björklund 2015) indicates that carbamazepine, in general, binds stronger than diclofenac, but this will depend on the specific composition of the matrix in question.

**Table 3:** Removal efficiency (%) of the six studied drugs at Osby and Kristianstad WWTP

Substance	Osby (grab)	Kristianstad (grab)	Kristianstad (24 h)	Skövde 2010	Umeå 2010	Uppsala 2010	Stockholm 2010	Henriksdal 2005–2009	Bromma 2005–2009
	This study	This study	This study	IVL	IVL	IVL	IVL	Stockholm Vatten	Stockholm Vatten
Atenolol	89	73	100	20	28	83	78	55	34
Bendroflumethiazide	100	89	67	NA	NA	NA	NA	NA	NA
Carbamazepine	84	97	48	−51	33	25	−20	7	−11
Diclofenac	81	38	12	−27	50	23	64	20	10
Furosemide	−34	44	−725	NA	NA	NA	NA	10	12
Oxazepam	76	89	34	−38	−2	31	−13	−6	15

Removal efficiencies are compared with those reported in the Swedish survey from 2010 of WWTPs (IVL 2011) covering the cities of Skövde, Umeå, Uppsala, and Stockholm and with data from the Swedish survey during 2005 to 2009 at Henriksdal and Bromma (Stockholm Vatten 2010)

*NA* data not available because the compound was not analysed in the study

Furosemide is a compound negatively charged at pH 7. Here our data, albeit with great variation, show a low removal efficiency (Table 3). The Swedish survey from 2010 did not include this compound (IVL 2011), whereas the survey from 2005 to 2009 reported removal efficiencies of 10 and 12 % for Henriksdal and Bromma, respectively. Large differences were seen in the Spanish study by Gros et al. (2010) where furosemide was removed to between 20 and 96 % with an average of 50 % (±59). Likewise, in Spain Rosal et al. (2010) observed a 60 % removal, whereas Castiglioni et al. (2006) reported the average removal for furosemide to be 8 % (range 0–17 %) during the Italian winter period and 54 % (range 15–62 %) in summer period. Our recent sorption data indicate a strong repulsion of the negatively charged furosemide from negatively charged sites on natural matrices just as previously described for diclofenac (Svahn and Björklund 2015). This, in combination with a low log P of 2.03 (Table 1), makes it amenable to pass through WWTPs. The removal occurring is most likely caused by other processes than chemical, e.g., biodegradation.

Oxazepam, being a neutral compound with a log P of 2.24 (Table 1), showed a removal between 34 and 89 % (Table 3). In the Swedish survey from 2010, removal ranged from −38 % (Skövde) to 31 % (Uppsala) (IVL 2011). Low removal efficiency was also seen in 2005–2009 at ≤15 % (Stockholm Vatten 2010). Equally, in the Czech Republic, Golovko et al. (2014) found a removal of −17 % during a 12-month period, whereas in France, Saussereau et al. (2013) found a removal of 7.5 %. Our sorption study shows that oxazepam sometimes shows a relatively high degree of sorption to matrices rich in organic matter (Svahn and Björklund 2015). Oxazepam carries no charge, which means that other forces are at play depending on the matrix characteristics.

### Environmental Concentrations and K_d_ Values

Brunkelstorp (SP1), a relatively pristine area far from any discharge sites or point sources (Fig. 1), showed no traces of the pharmaceuticals in the water phase. In Osbysjön (SP3), most pharmaceuticals were absent in the water phase as well as the sediment. Because this lake (SP3) is located at some distance from the WWTP, it is likely that the compounds have been diluted to lower than detection limits.

In *Vattenriket*, a potential source of pharmaceuticals is the old landfill of Kristianstad (SP4, Fig. 1). Four of the six compounds were identified, i.e., the two acidic pharmaceuticals furosemide and diclofenac as well as the more neutral compounds carbamazepine and oxazepam. Concentrations were 46, 192, 54, and 15 ng/L for the four compounds, respectively. The final covering of the landfill was performed in 2015 to decrease the infiltration of water, thereby decreasing the amount of impurities leaching into the River Helge Å. In the initial plans, Kristianstad municipality planned to cover the landfill with a mixture of sludge from the treatment plant, ash, and gravel to between 0.7 and 1.5 m. However, the County Administrative Board of Skåne rejected the proposal. Yet, during the summer of the year 2000, 5 ha of the landfill was covered with the mixture, and a conservative estimate shows that at least 17,500 m^3^ of sludge was added to the landfill. The measured concentrations of pharmaceutical residues might be derived both from the older landfill waste and the experiment with the recently deposited sludge. Notably, the content of diclofenac was as high in the leachate as in the 1500-m channel (SP6), which is described later in the text. In our previous study on Mallorca, we identified very high levels of furosemide (3840 ng/L) in one of the leachates, whereas diclofenac appeared at levels of between 22 to 40 ng/L (Rodríguez-Navas et al. 2013). Likewise, carbamazepine was quantified at levels of 52–123 ng/L, whereas oxazepam could not be detected. Concerning atenolol and bendroflumethiazide, the former reached levels of 237 ng/L, whereas the latter was not found. Without a doubt, landfill leachate is a source of pharmaceuticals to *Vattenriket* as well as other parts of Europe.

As seen in Fig. 1, the treated wastewater of Kristianstad town (SP5) flows into a 1500 m channel (SP6). The channel water is then pumped into lake Hammarsjön (SP7). The channel is characterized by slowly flowing water with a width of 2–3 m and a depth of approximately 1–2 m. The sampling point was located approximately 1400 m downstream of the WWTP outlet. With some exemptions, the concentrations in the channel were lower then those observed in the outlet water of Kristianstad WWTP, thus showing some process of removal whether biotic or abiotic (Figs. 3, 4). One process at play is sorption to the channel sediment. The concentration of atenolol reached >8000 ng/kg, which yielded a *K*_d_ value of 9.4 L/kg. *K*_d_ values for atenolol, ranging between 1.5 and 8.1 L/kg, were previously reported for river sediments by Yamamoto et al. (2009). Bendroflumethiazide was not found in the sediment, whereas carbamazepine reached levels >55,000 ng/kg with the latter compound yielding a *K*_d_ value of 125 L/kg. The sorption of psychoactive drugs has been studied by Stein et al. (2008). They found two *K*_d_ values of 1.7 and 12.3 L/kg for two different sediments, respectively. Logically, the sediment with the lowest percentage TOC of 0.74 % showed the lowest *K*_d_ value, whereas the greater *K*_d_ value corresponded to a TOC of 4.36 %. In this study, the sediment had a TOC value of almost 7.9 %. In our recent study, we observed strong correlations between TOC content and asymmetry factors in correspondence with these findings for natural sediments (Svahn and Björklund 2015). It should be noted, though, that Krascsenist et al. (2008) observed K_d_ values >50 L/kg for sediments with a TOC content of only 0.34 %, thus showing the complexity involved in describing pharmaceutical sorption. The complexity involved in the sorption mechanisms of drugs, such as tetracyclines (Scheytt et al. 2005), has also been addressed in other studies. Diclofenac had a concentration of >11.000 ng/kg in the channel giving a *K*_d_ value of 62 L/kg. In the study by Krascsenist et al. (2008), *K*_d_ values of approximately 4 L/kg were found for diclofenac. Although for carbamazepine, the TOC content could not explain the differences in *K*_d_ values, for diclofenac this is a reasonable explanation. We observed a 15 times greater *K*_d_ value, whereas the TOC value was 23 times greater. Furosemide had the highest *K*_d_ value of all compounds investigated, 2517 L/kg, as well as the highest measured concentrations at >350 µg/kg. No environmental studies were identified with which to compare our data. Finally, oxazepam had sediment concentrations exceeding 100 µg/kg and a *K*_d_ value of 402 L/kg. Stein et al. (2008) observed *K*_d_ values of 2.0 and 23.5 L/kg for the two investigated sediments with TOC values of 0.74 and 4.36 %, respectively.

As the final point, sampling was made in Lake Hammarsjön (SP7) at some distance from the outlet (150 m) of the channel water in the lake. For all pharmaceuticals, the concentrations in the lake were lower than those found in the channel (Figs. 3, 4). Although the lake water is somewhat stagnant and sorption might occur, this seems not to be the case. Only minute amounts of pharmaceuticals were found in the lake sediment, possibly due to a low organic content of only 0.43 %. Dilution is a more plausible explanation of the decreased concentrations in Lake Hammarsjön compared with the channel.

### Environmental Concentrations and Aquatic Toxicity

Comparing our measured environmental surface water concentrations with the current knowledge on ecotoxicological studies implies that the investigated pharmaceuticals could pose a risk toward aquatic organisms. Screening the literature for the most sensitive effects, we found a 21-day reproduction study with adult fathead minnow (*Pimephales promelas*) exposed to atenolol (Winter et al. 2008). Among 12 end points, Winter et al. found a no observable–effect concentration (NOEC) at 1.0 mg/L atenolol for a standardised condition index describing general physiological fitness and health as the most sensitive end point. Comparing this NOEC value with our highest measured environmental concentrations in Lake Hammarsjön (331 ng/L) and applying the appropriate assessment factor of 10 according to existing risk-assessment guidelines (VICH-GL38 2006), we found a risk quotient (RQ) at 0.003. Similarly for carbamazepine, observed at 161 ng/L in Lake Hammarsjön, the most sensitive end point, a NOEC value of 25,000 ng/L from a 7-day water flea reproduction study (*Ceriodaphnia dubia* [Ferrari et al. 2003]), yields an RQ of 0.06. Diclofenac was found at 119 ng/L in the same lake, and our literature study showed zebrafish (*Danio rerio*) in a 72-h embryo test (NOEC of 1.5 mg/L) as the most sensitive aquatic organism (van den Brandhof and Montforts 2010). Thus, the RQ for diclofenac is 0.0008. Furosemide was found at 100 ng/L in Lake Hammarsjön, and the most sensitive aquatic organism was also here the water flea (*C. dubia*) with a NOEC of 156 µg/L when considering population growth as the end point (Isidori et al. 2006). The resulting RQ value for furosemide is therefore 0.006. We observed oxazepam at 115 ng/L in the same lake. Chiffre et al. found a behavioural changes in Japanese medaka (*Oryzias latipes*) larvae exposed to oxazepam with a lowest observed–effect concentration (LOEC) at 10 µg/L (Chiffre et al. 2014). Consequently, the RQ for oxazepam is 0.24 when using an assessment factor of 20 for LOEC values. We observed 0.7 ng/L of bendroflumethiazide in Lake Hammarsjön; however, no usable ecotoxicological studies were available to risk assess this substance.

In summary, all single-substance risk quotients are <1; therefore, an immediate risk is unlikely. Yet no ecotoxicological studies were available considering the mixture effects of our targeted pharmaceuticals. Because mixtures of pollutants are likely to potentiate toxicological effects (e.g., Nielsen et al. 2015), there is a need to investigate this further.

## Conclusions

Osby and Kristianstad WWTPs, and to some extent leachate water from the Kristianstad Old landfill, are an undoubted source of the drug residues found in the aquatic environment of *Vattenriket*. Concerning Hanöbukten, it cannot be ruled out that River Helge Å downstream of Hammarsjön provides a variety of pharmaceutical residues. In our survey, all six investigated pharmaceuticals were found in Hammarsjön. We therefore propose an extended monitoring process southward including both pelagic and benthic samples on at least two occasions during the year. If conditions in the Hanöbukten bottom bay are similar to those prevailing in the channel, i.e., high organic content with low oxygen supply, it can not be excluded that drug residues are stored in the sediments over time. Recent research on oxazepam reveals a possible build up of this compound in lake sediments for decades under a business-as-usual scenario (Klaminder et al. 2015). Our risk assessment showed that adverse effects due to single-substance toxicity on fish populations harbouring the UNESCO Biosphere Reserve are not likely. However, cocktail effects from combinations of pharmaceuticals, metals, nutrients, or even metabolites or transformation products remain unsolved. In a coming study, we will analyse compounds according to the European Union Watch List (EU Decision Watch List 2015) as well as compounds recently proposed by the Swedish Medical Products Agency (Läkemedelsverket 2015) to improve our knowledge of the chemical cocktail entering Hanöbukten as proposed by the Swedish Agency for Marine and Water Management (Hanöbukten 2013).
